# Transcriptomic Changes and Regulatory Networks Associated with Resistance to Mastitis in Xinjiang Brown Cattle

**DOI:** 10.3390/genes15040465

**Published:** 2024-04-07

**Authors:** Dan Wang, Haiyan Yang, Shengchao Ma, Tingting Liu, Mengjie Yan, Mingming Dong, Menghua Zhang, Tao Zhang, Xiaoxue Zhang, Lei Xu, Xixia Huang, Hong Chen

**Affiliations:** 1 College of Animal Science, Xinjiang Agricultural University, Urumqi 830091, China; wangdan01100330@163.com (D.W.); shengchaomasicau@163.com (S.M.); liutingting1109@126.com (T.L.); y13095066028@163.com (M.Y.); dongmingming@xdmy.com (M.D.); zhangmenghua810@126.com (M.Z.); z13319734240@163.com (T.Z.); zhangxiaoxue0726@163.com (X.Z.); q609468041@sina.com (L.X.); 2 College of Animal Science and Technology, Northwest A&F University, Yangling, Xianyang 712100, China; yanghaiyanzsl@163.com

**Keywords:** Xinjiang brown cattle, mastitis resistance, RNA-seq, ceRNA interaction network

## Abstract

Xinjiang brown cattle are highly resistant to disease and tolerant of roughage feeding. The identification of genes regulating mastitis resistance in Xinjiang brown cattle is a novel means of genetic improvement. In this study, the blood levels of IL-1β, IL-6, IL-10, TNF-α, and TGF-β in Xinjiang brown cattle with high and low somatic cell counts (SCCs) were investigated, showing that cytokine levels were higher in cattle with high SCCs. The peripheral blood transcriptomic profiles of healthy and mastitis-affected cattle were constructed by RNA-seq. Differential expression analysis identified 1632 differentially expressed mRNAs (DE-mRNAs), 1757 differentially expressed lncRNAs (DE-lncRNAs), and 23 differentially expressed circRNAs (DE-circRNAs), which were found to be enriched in key pathways such as PI3K/Akt, focal adhesion, and ECM-receptor interactions. Finally, ceRNA interaction networks were constructed using the differentially expressed genes and ceRNAs. It was found that keynote genes or mRNAs were also enriched in pathways such as PI3K-Akt, cholinergic synapses, cell adhesion molecules, ion binding, cytokine receptor activity, and peptide receptor activity, suggesting that the key genes and ncRNAs in the network may play an important role in the regulation of bovine mastitis.

## 1. Background

Milking of dairy cattle has been practiced since at least 3100 BCE [1]. Manual milking allows easy monitoring of milk and mammary abnormalities due to close contact, but little is known about the etiology or treatment of mastitis [2]. Mastitis remains one of the most common complex diseases found on cattle farms, and the high incidence of mastitis and the prevalence of sub-clinical or recessive mastitis not only adversely affect the production and quality of the milk but are also associated with reduced fertility and a shorter productive life span of the cows, as well as increased treatment costs, thus seriously limiting the economic benefits of dairy cattle farming [3]. Bar et al. analyzed the daily production of 10,380 lactating cows in five large, high-producing dairy cattle herds and found that many cows were more productive before developing the disease than unaffected individuals, that within two months of the mastitis diagnosis, the cows lost 164 kg of milk in the first lactation and 198 kg in the second lactation, and that production did not recover [4]. Additionally, the microbial communities in the milk can also affect the development of the immune system in calves [5,6]. Currently, selection for mastitis resistance is generally based on the SCC, udder depth, and anterior udder attachment [7], with correlation coefficients between SCC and mastitis ranging from 0.60 to 0.90 [8]. According to the International Dairy Federation (IDF), an SCC of 500,000 cells/mL represents the threshold of recessive mastitis, while an SCC value > 1,000,000 cells/mL indicates that the udder is infected and damaged. Due to the skewed distribution of the SCC data, SCC was converted to a somatic cell score (SCS) for genetic analysis. 

Xinjiang brown cattle are the first dual-purpose cattle breed raised in China with independent intellectual property rights. The breed shows superior milk quality, food tolerance, and disease resistance compared with other breeds. Numerous phenotypic data on Xinjiang brown cattle have been collected, and comparative analysis of dairy herd improvement (DHI) records has shown that under the same feeding and management conditions, the SCCs of Xinjiang brown cattle are significantly lower than those of Holstein cattle, suggesting the potential usefulness of investigating mastitis resistance in this breed [9]. Indeed, the mastitis-related traits of Xinjiang brown cattle have been thoroughly investigated. Zhou et al. used the results of GeneSeek GGP Bovine 150 K chip detection and performed a genome-wide association study (GWAS) that identified three single-nucleotide polymorphisms (SNPs) closely associated with the somatic cell scores (SCSs) of Xinjiang brown cattle [10]. A further study on correlations between insertion–deletions (InDels) in the fragile histidine triad (*FHIT*) gene and lactation traits in Xinjiang brown cattle revealed significant associations between eight InDels in the gene and lactation traits [11], while an investigation using pyrosequencing showed that methylation of the promoter regions of the *FHIT* gene and protein inhibitor of activated *STAT1* and *PIAS1* genes in mastitis-affected cows were higher and lower, respectively, than those in the control group [12]. Nevertheless, the differences in gene expression levels associated with mastitis in Xinjiang brown cattle have not been fully analyzed at the transcriptome level.

With the rapid development of high-throughput technology, data from multiple “omics” investigations has been reported, resulting in the identification of new phenotypes and facilitating the analysis of the underlying genetic mechanisms associated with key traits [13]. For instance, Shangraw et al. infused lipopolysaccharide into the udder to elicit an inflammatory response and performed RNA-seq to gain a deeper understanding of the mechanisms controlling the spatiotemporal responses of local mastitis in lactating dairy cattle. This led to the identification of 3088 and 1644 differentially expressed genes (DEGs) at 3 and 12 h, respectively, after infusion, of which *NFKBIA*, *TNFAIP3*, and *VCAM1* were found to be hub genes [14]. Liu et al. constructed a cattle genotype-tissue expression map (CattleGTEx) based on 7180 public RNA-seq sequence fragments, revealing hundreds of thousands of genetic correlations with gene expression and variable splicing in 23 different tissues and identifying an association between mammalian mitochondrial ribosomal protein L45 (*MRPL45*) and SCS [15]. Turk et al. analyzed the systemic inflammation and oxidative stress response in dairy cattle with sub-clinical and clinical mastitis and found that both A3-1 and complement factor H were differentially expressed in individuals with sub-clinical mastitis [16]. It is apparent that mastitis is a complex disease, and its development is not associated with single factors. Therefore, the availability of multilevel “omics” data provides an opportunity to analyze mastitis resistance in Xinjiang brown cattle and thus improve selection and breeding.

The concept of competing endogenous RNA (ceRNA) was first proposed in 2011 by a research team from Harvard Medical School [17]. The term refers to an endogenous RNA species that has binding sites for various microRNAs (miRNAs), thus competing for miRNA binding and degradation. CeRNAs can include lncRNAs, circRNAs, and post-transformationally modified protein-coding RNAs. Binding between mRNAs and miRNAs can alter the translation of the mRNA. RNAs can communicate with each other through miRNA and miRNA-binding sequences (MREs). The greater the number of shared MREs, the higher the level of “communication”; the 3’UTRs of mRNA contain MREs, which can regulate the RNA molecule itself in cis and may also regulate the levels of miRNA and other RNAs in trans. LncRNAs are known to be involved in a variety of biological processes, such as transcriptional regulation, RNA processing, and the control of protein stability. LncRNAs can regulate gene expression and function through a variety of mechanisms, such as RNA interference, epigenetic modifications, signaling, and the assembly of nucleoplasmic transcription complexes [18]. They are also involved in the development and progression of various diseases [19] and have been implicated in mastitis [20]. The first miRNA (Lin-4) was discovered in *Caenorhabditis elegans* in 1993 [21], and subsequent research on mammalian miRNAs has developed rapidly. Mature miRNA binds to the RNA-induced silencing complex (RISC) to form a functional RNA complex. The miRNA searches for complementary target mRNAs in RISC complexes based on their complementary pairing and, after binding, inhibits the expression of the target mRNA through RNAi effects or other mechanisms. miRNAs form complexes with Argonaute proteins to form RISCs, and their complementary pairing with target mRNA sequences regulates mRNA translation or degradation [22,23].

Mastitis resistance is a complicated trait, and its regulatory mechanism is very complex. There are still many important ncRNAs that have not been explored and whose functions are yet to be investigated. This is especially true of research on ncRNAs associated with mastitis resistance. Xinjiang brown cattle represent a characteristic germplasm resource in Xinjiang, China, and show good tolerance and disease resistance, with superior milk quality to many other varieties of dairy cattle. The transcriptomic analysis of whole blood can be used to study immunobiology and identify new biomarkers [24], thus providing technical and theoretical references for the mining of molecular markers for mastitis resistance. In this study, Xinjiang brown cattle were selected for investigation, and healthy cattle and cattle with clinical mastitis were selected using a combination of SCC levels and the detection of pre-pathogenic bacteria and clinical diagnosis. From the perspective of transcriptional regulation, the mRNA, lncRNA, and miRNA expression profiles associated with mastitis resistance in Xinjiang brown cattle were constructed using the results of RNA-seq, and key mRNA and ncRNA species were identified using bioinformatics tools to construct the ceRNA interaction network. Subsequently, GO and KEGG enrichment analyses were performed on the network to determine the ceRNA signaling axes that might be involved in Xinjiang brown cattle mastitis and provide a comprehensive analysis and explanation of the underlying genetic mechanism associated with mastitis in Xinjiang brown cattle, together with the search for key molecular markers.

## 2. Materials and Methods

### 2.1. Animal Selection and Sample Collection

The experimental animals were provided by the Xinjiang brown cattle breeding farm (Urumqi breeding farm, Urumqi, China) in this study. Six healthy Xinjiang brown cattle (the control group, SCC ≤ 200,000 cells/mL) and six Xinjiang brown cattle with clinical mastitis (the clinical mastitis group, SCC ≥ 5,000,000 cells/mL) were selected. These cattle were maintained under the same feeding management level, with the same lactation period and parity, had DHI production performance data, and were without any other disease. The California Mastitis Test (CMT) showed that six Xinjiang brown cattle (the clinical mastitis group) had at least one udder area of +++/++ and were thus diagnosed with clinical mastitis.

Blood samples were collected from the tail veins of 12 Xinjiang brown cattle. For this, 2.5 mL of whole blood was collected using a PAXgene Blood RNA Tube for total RNA extraction, and all whole blood samples were stored at −80 °C. Then, 5 mL of blood was collected into tubes (without anticoagulant), stood for more than 5 min, and centrifuged at 3500 rpm for 5 min. Then, serum was collected and stored at −80 °C for cytokine detection. 

### 2.2. ELISA Measurements

Levels of IL-1, IL-6, IL-10, TNF-α, and TGF-β in the peripheral blood of cattle in the high- and low-SCC groups were measured using the Bovine Interleukin 1β (IL-1β), Bovine Interleukin 6 (IL-6), Bovine Interleukin 10 (IL-10), Bovine Tumor Necrosis Factor α (TNF-α), and Bovine Transforming Growth Factor β (TGF-*β*) ELISA assay kits (Jiancheng, Nanjing, China). Then, data were analyzed and compared using t-Student statistical analysis in SPSS version 19.0 (IBM Corp., Armonk, NY, USA) and GraphPad Prism 8.0.1 (San Diego, CA, USA). *p*-values < 0.05 were considered significant and were marked with an asterisk (*).

### 2.3. RNA Extraction and Testing

Total RNA was extracted from the blood samples using the PAXgene Blood RNA Kit. The purity and concentration of the RNA were assessed using agarose gel electrophoresis and an Agilent 2100 bioanalyzer (Santa Clara, CA, USA).

### 2.4. Library Construction and Sequencing

Ribosomal RNA was separated from the total RNA and fragmented into 250–300 bp fragments using RNase R. First-strand cDNA was synthesized using the fragmented RNA as a template and random oligonucleotides as a primer. RNase H was used to degrade the RNA strand, and the DNA polymerase I system was used to synthesize the second strand of cDNA using dUTP, dATP, dGTP, and dCTP as substrates. End repair was performed on the double-stranded cDNA, an A-tail was added with a sequencing junction, and cDNA of 350–400 bp in length was isolated using AMPure XP beads. The second strand of the U-containing cDNA was degraded using the USER enzyme. PCR amplification was performed to obtain the cDNA library. Qubit was diluted after initial quantification (1 ng/μL), Agilent 2100 was used to determine the insert size (250–300 bp), and the effective concentrations (>2 nM) were quantified by qPCR. After quality control, cDNA libraries were sequenced using the Illumina PE150 platform.

### 2.5. Data Quality Control and Comparison

The raw data obtained from the Illumina PE150 platform was converted to sequenced reads by CASAVA base identification and stored as FASTQ files. Reads with adapters or N > 0.002 and with low-quality bases containing more than 50% were eliminated. Q-Phredreads was used to assess the quality of the bases, and the sequencing error rate was calculated using the following formula: Q-Phred = −10log_10_(e). After filtering the raw data and checking the sequencing error rate and distribution of GC content, the resultant clean reads were used for subsequent analyses.

RNA-Seq data were mapped using STAR v2.7.11 [25], Tophat v2.1.1 [26], and Hisat 2 v2.2.0 [27]. The clean reads were mapped to the cattle reference genome (ARS-UCD1.2) to determine the positions and proportions of the reads in the genome (exons, introns, and intergenic regions).

### 2.6. Transcript Splicing and Screening

First of all, cuffmerge [28] was used to combine the spliced transcripts from each sample and remove transcripts with uncertain strand orientation and length < 200 nt. Then, Cuffcompare was used for comparison against data from databases, to filter out the annotated transcripts contained in the database, and to predict the coding potential of the identified transcripts, ultimately yielding novel lncRNAs and mRNAs. The splicing and screening criteria were transcript exon number ≥ 2 and transcript length > 200 nt. CPC2 v2.0 (http://cpc2.cbi.pku.edu.cn, URL: May 2022), Pfam v36.0 (http://pfam.xfam.org/ URL: May 2022), and CNCI (https://github.com/www-bioinfo-org/CNCI, URL: May 2022) were used for the prediction of the coding potential, and the intersection of the software results was termed novel mRNA. The lncRNAs were compared with the mRNAs to identify exon numbers, transcript lengths, differences in open reading frames, and features.

### 2.7. Quantitative and Differential Analysis

StringTie v2.1.5 (https://ccb.jhu.edu/software/stringtie/, URL: May 2022) was used to quantify the known transcripts as well as predict the lncRNAs, mRNAs, and unclassified transcripts after sequence alignment, splicing, and filtering. The gene expression levels from the RNA-seq data were expressed as fragments per kilobase of transcript per million mapped reads (FPKM), and edgeR was used to test the significance of the quantitative results for differential expression. Additionally, the *p*-value or the corrected *p*-value was used to test the significance level.

### 2.8. GO and KEGG Enrichment Analysis

Gene Ontology (GO) and Kyoto Encyclopedia of Genes and Genomes (KEGG) enrichment analyses of DEGs and target genes of DE-lncRNAs were performed using ClusterProfiler [29] and KOBAS v3.0 (http://kobas.cbi.pku.edu.cn, URL: May 2022). *p* < 0.05 was used as the criterion to identify significant GO terms or KEGG pathways. The GO and KEGG enrichment results were visualized using the ggplot in the R package.

### 2.9. Prediction of lncRNA Target Genes

Co-location and co-expression analyses were conducted simultaneously. The threshold of co-location was set at 100 kb upstream and 100 kb downstream of the lncRNA, according to the co-location regulation mechanism.

### 2.10. RT-qRCR

DE-mRNAs (*OX1*, *ROPN1*, *NRXN2*, *TLR4*, *CDH9*, *C1R*, *WDR72*, and *SV2B*) were selected from the sequencing results, and the primers were designed by NCBI. The primer sequences are shown in Table S1. RNA was reverse-transcribed into cDNA using the Evo M-mlV RT Kit (Accurate Biology, Changsha, China, AG11711) by RT-qRCR, and qPCR was performed using the 2 × Taq SYBRGreen^®^ qPCR Mix (Innovagene, Changsha, China), with a 10 μL reaction system consisting of 5 ng cDNA, 0.5 μL each of the upstream and downstream primers, 5 μL SYBRGreen Mix, and 3 μL ddH_2_O. The relative expression was calculated using the 2^−ΔΔCt^ method. *GAPDH* was used as a reference gene. Gene expression was analyzed in each of the 12 Xinjiang brown cattle to verify the reliability of the DEGs identified by RNA-seq.

### 2.11. Establishment of ceRNA Networks

LncRNA-target gene pairs with the same miRNA binding sites were identified, and lncRNA-miRNA-mRNA regulatory relationships were assessed using the lncRNA as the decoy, the miRNA as the core, and the mRNA as the target, thus constructing a ceRNA-based regulatory network. Finally, the interaction network based on the lncRNA-miRNA-mRNA regulatory network was constructed and visualized using Cytoscape 3.9.0.

## 3. Results

### 3.1. Serum Cytokine Levels in the High- and Low-SCC Xinjiang Brown Cattle

**Figure 1:**
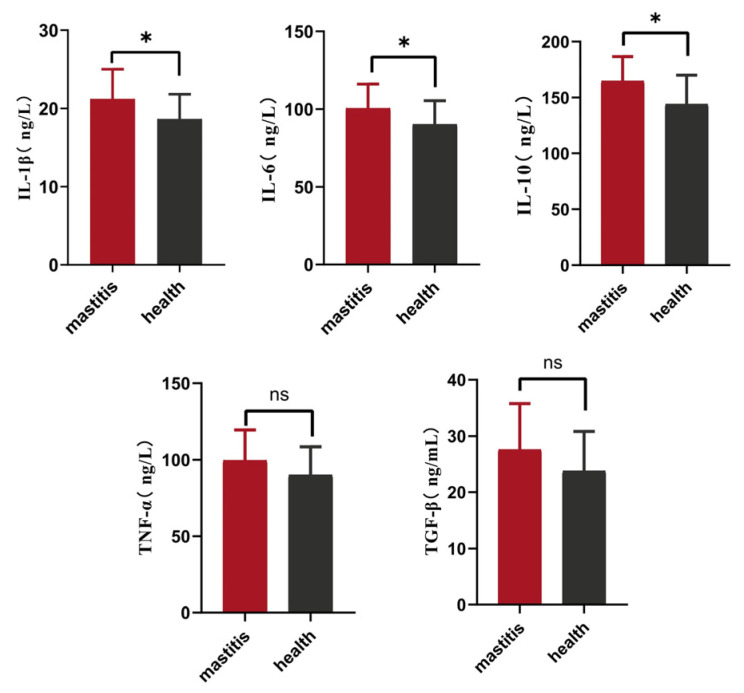
Serum cytokine levels in the high- and low-SCC groups. *: *p* ≤ 0.05, ns: not significant.

The mean levels of IL-1β, IL-6, IL-10, TNF-*α*, and TGF-*β* in the high-SCC group were 20.84, 98.36, 161.84, 97.27, and 26.90 ng/mL, respectively, which were significantly higher than those in the sera of the control group (low-SCC group) (*p* ≤ 0.05) (Figure 1).

### 3.2. Sequencing and Characterization of lncRNAs

**Figure 2:**
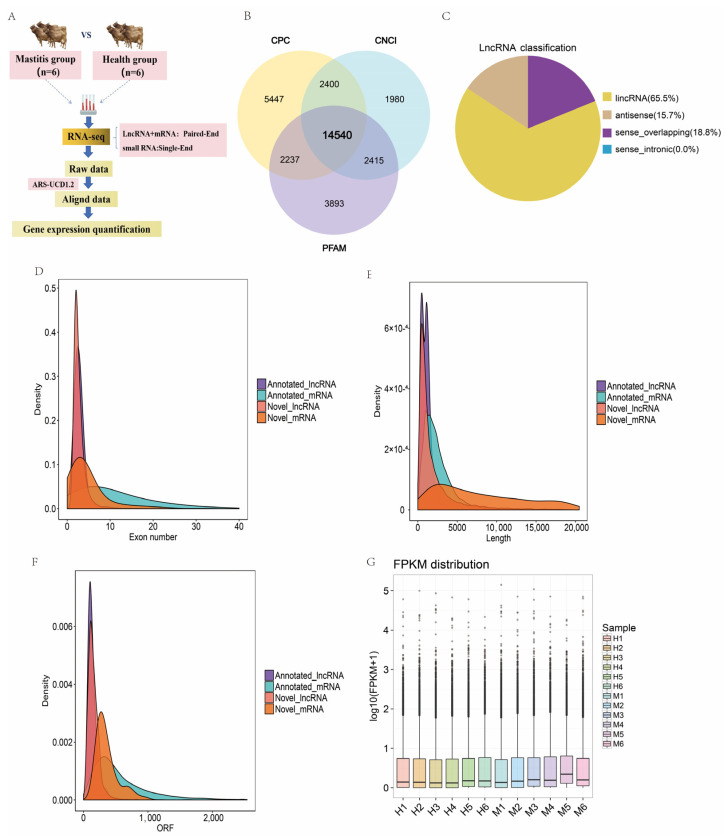
Sequencing and characterization of lncRNAs and mRNAs. (**A**) Workflow. (**B**) Coding potential filter; (**C**) LncRNA-type distribution map. (**D**) Exon number density distribution map of lncRNAs and mRNAs. (**E**) Length comparison density distribution map of lncRNAs and mRNAs. (**F**) Distribution of open reading frame densities of lncRNAs and mRNAs. (**G**) Distribution of expression levels in each sample.

Figure 2A illustrates the sequencing and characterization processes in the two groups of Xinjiang brown cattle. The quality of the RNA is shown in Table S2. Raw data were filtered, removing reads with junctions, unknown bases, and those of low sequencing quality. The base error rate was less than 0.04%, and the percentage GC content ranged from 46.8 to 50.04%, which was similar to that of the cattle genome. The total sequencing data content of a single sample was 12.07–14.14 G, with a Q20 higher than 97% and a Q30 higher than 92% (Table S3). Overall, the quality of the sequencing data was excellent, thus providing a foundation for the subsequent data analysis. The 12 RNA-seq clean data were mapped to the reference genome; an average of 94.27% of the reads aligned with the reference genome (Table S4), and 90.95% of the reads were found to have a unique mapped genome position (uniquely mapped). The identification of lncRNAs after transcript assembly yielded a total of 90,635 transcripts, which were then analyzed in terms of structural characteristics and whether they had protein structural domains and coding potential. This identified 14,540 transcripts with no coding ability (Figure 2B), as well as 2198 known lncRNAs, 13,075 novel lncRNAs, 37,518 known mRNAs, and 820 novel mRNAs. Based on the positional relationship between the HUGO Gene Nomenclature Committee (HGNC) information (https://www.genenames.org/, URL: May 2022) and known mRNAs, the identified lncRNAs were categorized into three types. Of these, 65.5% of the lncRNAs were located in the intergenic region, 18.8% overlapped with one or more exons of protein-coding genes of the same strand (exon overlapping), and 15.7% overlapped with one or more exons of protein-coding genes of the opposite strand (antisense) (Figure 2C). A comparison and analysis of the exon numbers and transcript lengths of the 13,075 novel lncRNAs and 820 mRNAs showed that the distribution type and structure of the novel mRNAs and LncRNAs were consistent with general characteristics (Figure 2D–F). The known transcripts, which were compared, spliced, and screened in the previous stage, predicted the novel lncRNAs, novel mRNAs, and unclassified transcripts, which were quantified in terms of expression levels, and the FPKM values of the 12 sequencing data were calculated. Figure 2G shows a box plot of the distribution of the expression levels in the individual samples, showing that the levels of expression tended to be consistent in all samples (Figure 2G).

### 3.3. DE-lncRNAs

**Figure 3:**
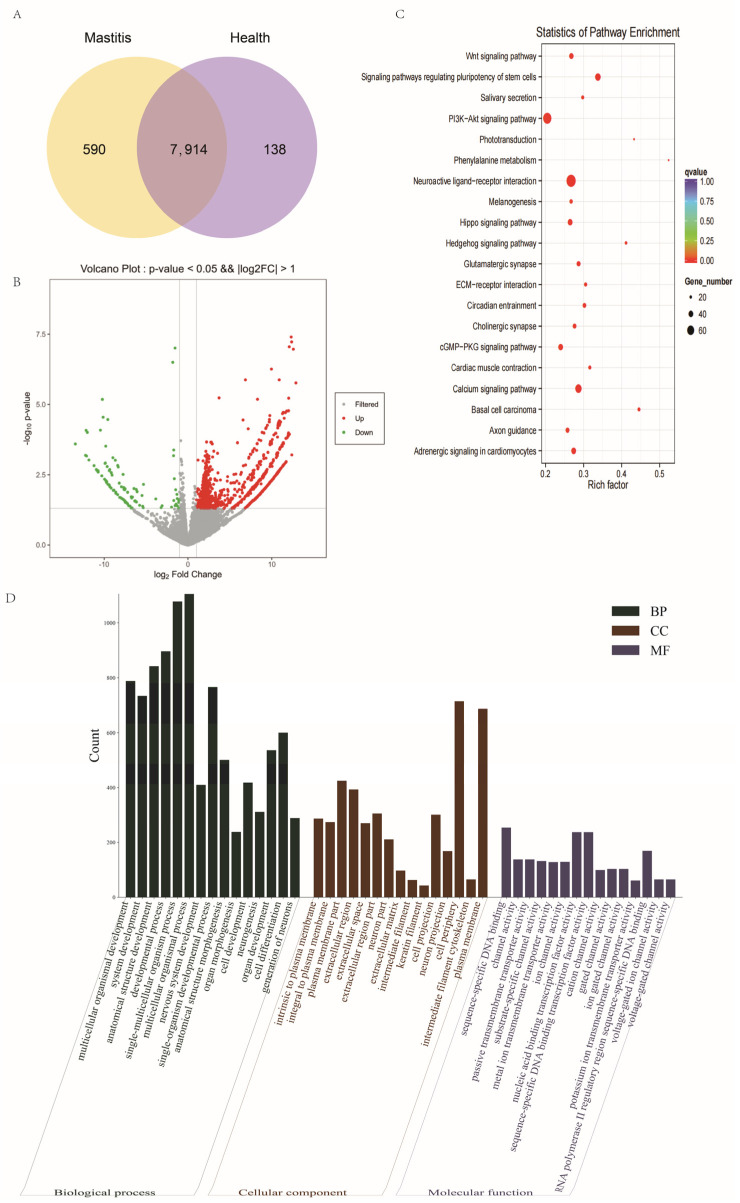
DE-lncRNAs; (**A**) LncRNAs expressed in two groups. (**B**) The volcano map of DE-lncRNAs. (**C**) KEGG enrichment analysis of DE-lncRNA target genes. (**D**) GO enrichment analysis of DE-lncRNA target genes.

DE-lncRNAs between the clinical mastitis and control groups were compared to find key lncRNAs that may affect the occurrence and development of Xinjiang brown cattle mastitis. A total of 7914 lncRNAs were identified in two groups, of which 1757 were differentially expressed at the transcript level in the mastitis vs. healthy comparison, with 1537 up-regulated and 220 down-regulated (Figure 3B). KEGG enrichment analysis showed that the target genes of the lncRNAs were mainly enriched in signaling pathways such as neuroactive ligand–receptor interactions, the PI3K-Akt signaling pathway, and calcium signaling pathways, among others (Figure 3C), which laid the foundation for the identification of the novel hub genes. The results of GO enrichment analysis revealed that the target genes of the DE-lncRNAs were mainly enriched in multicellular organismal development, system development, multicellular organismal processes, intrinsic to the plasma membrane, cell development, sequence-specific DNA-binding RNA polymerase II transcription factor activity, and central nervous system development (Figure 3D).

### 3.4. DE-mRNAs

**Figure 4:**
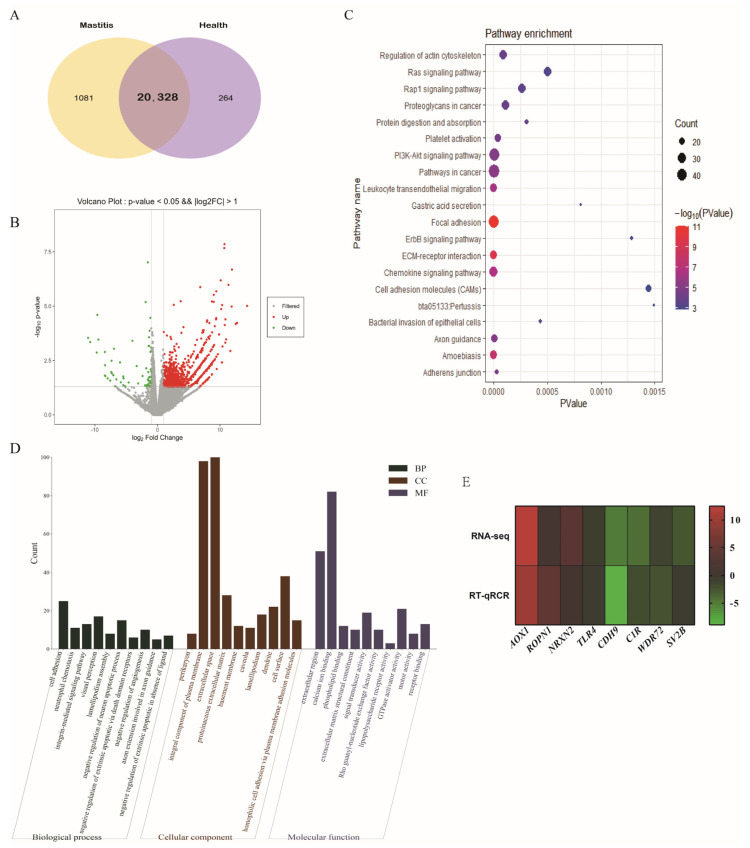
DE-mRNAs. (**A**) mRNAs expressed in two groups. (**B**) The volcano map of DE-circRNAs. (**C**) KEGG enrichment analysis of DE-mRNAs. (**D**) GO enrichment analysis of DE-lncRNA target genes. (**E**) Verification of RNA-seq results by RT-qPCR.

To explore the hub genes that may affect the occurrence and development of mastitis in Xinjiang brown cattle, the DE-mRNAs between the control and clinical mastitis groups were compared. Overall, 20,328 mRNAs were detected in two groups of Xinjiang brown cattle (Figure 4A). Of these, 1632 mRNAs were found to be differentially expressed between the two groups, with 1055 showing up-regulation and 577 showing down-regulation (Figure 4B). KEGG enrichment analysis (Figure 4C) revealed that the DE-mRNAs were mainly enriched in pathways associated with focal adhesion, ECM-receptor interactions, chemokine signaling, and other important signaling pathways. GO enrichment analysis (Figure 4D) revealed enrichment in integrin-mediated signaling pathways, cell adhesion, neutrophil chemotaxis, the extracellular space, and other GO terms. Eight DE-mRNAs were randomly selected for verification by RT-qRCR. As shown in Figure 4E, the RT-qPCR (left) detection of the mRNA expression pattern was consistent with the trend of the RNA-seq (right) data, demonstrating that the RNA-seq data were reliable.

### 3.5. Identification and Structural Analysis of circRNAs

**Figure 5:**
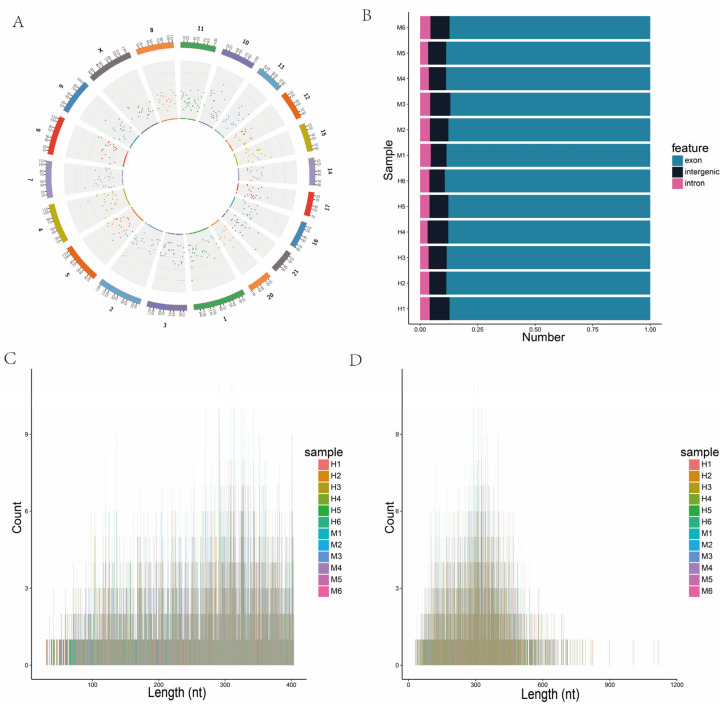
Analysis of circRNA characteristics. (**A**) Circos diagram of the density distribution of circRNAs on chromosomes. (**B**) Sources of circRNAs. (**C**) Length distributions of circRNAs. (**D**) Length distributions of circRNAs.

The circRNAs in the peripheral blood were identified using find_circ [30] and CIRI [31]. The expression of the identified circRNAs was further analyzed. The density distribution of all circRNAs on chromosomes is shown in Figure 5A. The circRNAs are derived from either exon or intron splicing, with some from intergenic regions (Figure 5B). Here, 88.1% of the circRNAs identified in the peripheral blood were from exon splicing, 7.9% from intron splicing, and 4% from intergenic region splicing. The lengths of the circRNAs were mainly between 100 and 400 bp, which was consistent with the general characteristics of circRNA (Figure 5C,D).

### 3.6. DE-circRNAs

**Figure 6:**
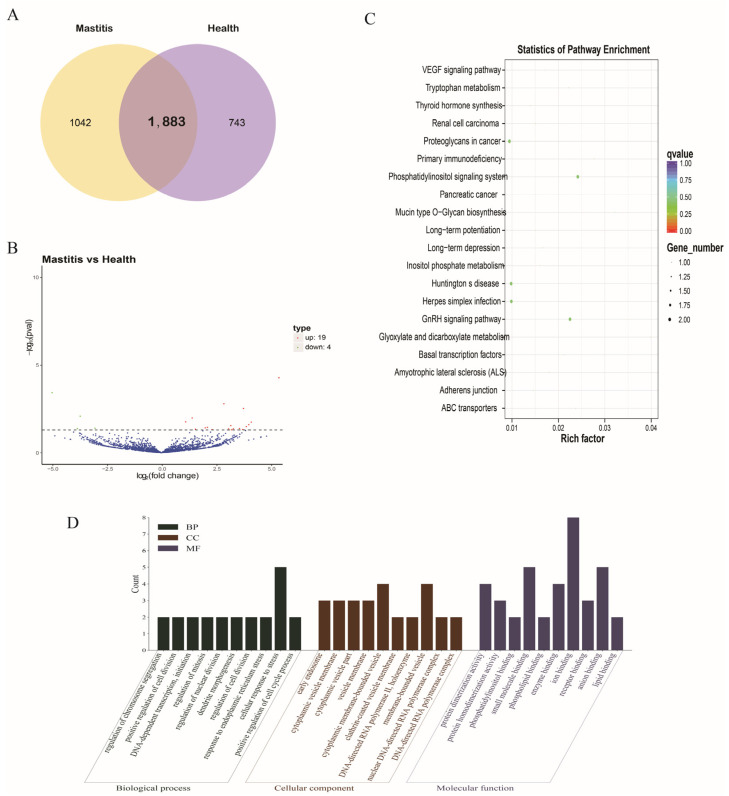
DE-circRNAs. (**A**) circRNAs expressed in the two groups. (**B**) Volcano map of DE-circRNAs. (**C**) KEGG enrichment analysis of DE-circRNA target genes. (**D**) GO enrichment analysis of DE-circRNA target genes.

The expression of circRNAs in each sample was analyzed and normalized by TPM. A total of 1883 circRNAs were found to be expressed in two groups, while 719 circRNAs were predicted to have no coding ability, as shown by CPC2, CNCI, and Pfam (Figure 6A). A total of 23 DE-circRNAs were identified (Figure 6B), of which 19 were up-regulated and 4 were down-regulated. After identifying the DE-circRNAs in each group, the circRNAs were annotated to their parental genes, and GO and KEGG enrichment analyses were performed on the parental genes. These were found to be significantly associated with pathways such as proteoglycans in cancer, the phosphatidylinositol signaling system, and the GnRH signaling pathway (Figure 6C), and were enriched in GO terms such as regulation of chromosome segregation, antigen processing and presentation of exogenous peptide antigens via MHC, positive regulation of cell division, the TAP complex, inositol-1, and 4,5-trisphosphate 5-phosphatase activity.

### 3.7. CircRNA-miRNA-mRNA Regulatory Network

**Figure 7:**
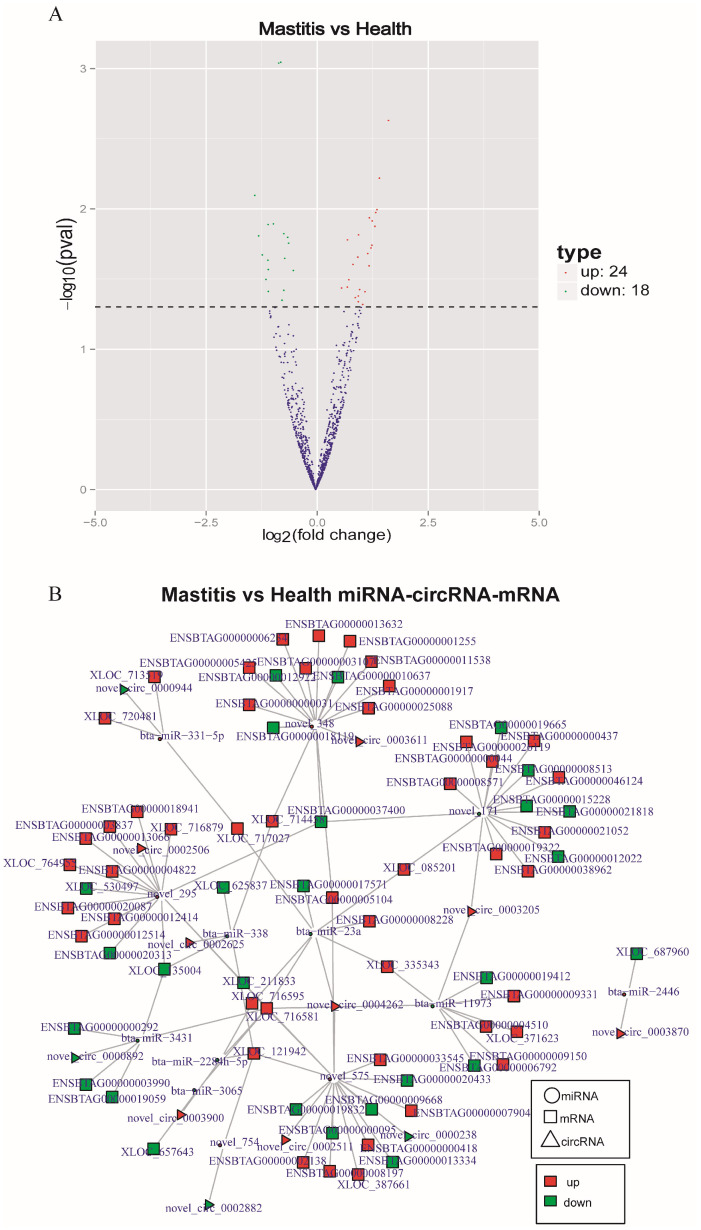
CircRNA-miRNA-mRNA network. (**A**) Differentially expressed miRNAs between healthy Xinjiang brown cattle and those with clinical mastitis. (**B**) circRNA-miRNA-mRNA interaction networks.

A total of 24 up-regulated and 18 down-regulated miRNAs were identified (Figure 7A). Cytoscape visualization of the circRNA-miRNA-mRNA network showed a total of 12 circRNA nodes (triangles), 13 miRNA nodes (circles), 119 mRNA nodes (squares), and 163 connections (Figure 7B), of which novel circ_0004262, novel circ_0003205, novel circ_0002625, and other circRNAs, novel 171, novel 348, novel 575, and other miRNAs, *XLOC_716581*, ENSBTAG00000037400 (*TNRC18*), ENSBTAG00000005104 (*MGAT5B*), and other mRNAs were located at the core of the interaction network, suggesting that the core nodes in the network might regulate the occurrence and development of Xinjiang brown cattle mastitis.

### 3.8. Regulatory lncRNA-miRNA-mRNA Network

**Figure 8:**
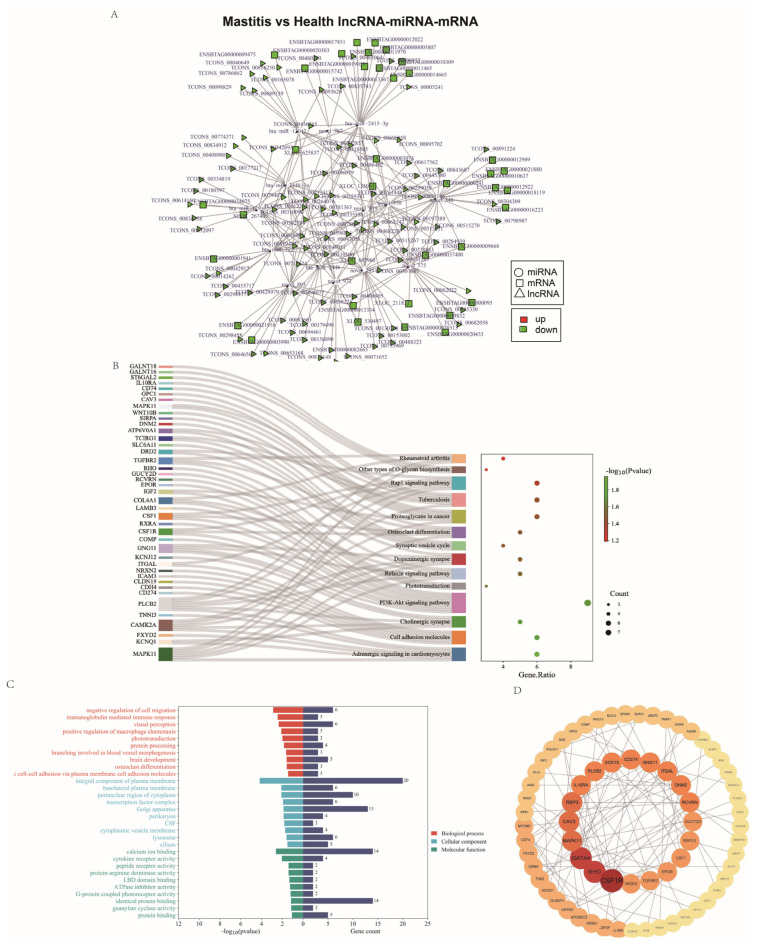
Key mRNAs in the lncRNA-miRNA-mRNA interaction networks. (**A**) LncRNA-miRNA-mRNA interaction networks. (**B**) KEGG enrichment analysis of mRNAs in the lncRNA-miRNA-mRNA network. (**C**) GO enrichment analysis of mRNAs in the lncRNA-miRNA-mRNA network. (**D**) Protein–protein interaction network and identification of hub genes.

Based on the common RNA data, the lncRNA (down-regulated)-miRNA (up-regulated)-mRNA (down-regulated) ternary interaction network was constructed and visualized using Cytoscape v3.9.0. A total of 1177 lncRNA nodes (triangles), 35 miRNA nodes (circles), 795 mRNA nodes (squares), and 4053 connecting lines formed the lncRNA-miRNA-mRNA network (Figure 8A), of which TCONS_00211035, TCONS_00114426, TCONS_00612301, and other lncRNAs; bta-miR-2415-3p, bta-miR-3431, bta-miR-2904, and other miRNAs; *XLOC_716581*, *HABP2*, *XLOC_121942*, and other mRNAs were located at the core of the interaction network. A lncRNA-miRNA-mRNA network was then constructed using the down-regulated expression trends (Figure 8A), in which there were 112 lncRNA nodes, 14 miRNA nodes, 107 mRNA nodes, and 408 connecting lines, of which TCONS_00047055, TCONS_00612301, TCONS_00062142, and other lncRNAs; novel 575, bta-miR-2415-3p, novel 972, and other miRNAs; and *SLC16A6*, *CSF3R*, *XLOC_135004*, and other mRNAs formed the core of the network, suggesting that these core nodes might regulate the occurrence and development of Xinjiang brown cattle mastitis. Additionally, KEGG and GO enrichment analyses of mRNAs in the lncRNA-miRNA-mRNA network (Figure 8B,C) revealed that genes in the network were mainly enriched in cancer pathways, the PI3K-Akt signaling pathway, cell adhesion molecules, negative regulation of cell migration, immunoglobulin-mediated immune responses, and other key terms. The network analysis showed that the *CSF1R*, *RHO*, *RCVRN*, *CAV3*, and *GATA4* genes were core nodes, suggesting that these genes could serve as candidate molecular markers for mastitis (Figure 8D).

## 4. Discussion

Inflammation is a protective response mounted by an organism against pathogens, infection sources, tissue damage resulting from infections, chemical stimuli, and other traumatic injuries [2]. It involves the coordinated communication of multiple immune cells and blood vessels. Host responses generate complex molecular signals that activate or modify immune cells and other cells [32]. Pro-inflammatory cytokines (e.g., IL-1β, IL-6, and TNF-α) are secreted mainly by macrophages, while the anti-inflammatory cytokines include IL-10 and TGF-β. When pathogens invade the mammary glands of cows, the consequent release of pro-inflammatory cytokines can activate the innate and adaptive immune systems, inducing an inflammatory response in the tissue, while anti-inflammatory cytokines are mainly involved in the reduction of inflammation after the elimination of the pathogens, so that the organism can return to a normal immune and physiological state [33]. Pro- and anti-inflammatory cytokines are key factors in the inflammatory responses associated with damage repair in Xinjiang brown cattle, and the collaborative and dynamic balance between these cytokines is an important contributor to the development of mastitis and its subsequent recovery and repair. Breast epithelial cells have multiple bacterial sensors that produce inflammatory mediators and local defense responses [34]. In the udder, the blood–milk barrier prevents the uncontrolled mixing of blood and milk components, thus maintaining an osmotic gradient that draws water into the udder secretions. In mastitis, the permeability of the blood–milk barrier increases, which is reflected in the transfer of blood components to the milk and vice versa. Changes in the immune status of an animal can alter its level of tolerance to environmental stimuli as well as its level of milk production, as in the case of cows. The degree of stimulation can be reflected by measuring the levels of cytokines [35]. The levels of the five inflammatory cytokines in the peripheral blood of Xinjiang brown cattle were found to be higher than those in the blood of the control group, and the levels of the pro-inflammatory cytokines IL-1β, IL-6, and IL-10 were significantly higher than those in the cows of the control group. Indeed, the measurement of cytokine levels could assess the difference in immune status between the clinical mastitis group and the control group. Numerous cytokines act at both local and systemic levels during the onset, progression, and resolution of inflammation. They provide relatively short-range communications between cellular immune components, thus linking the innate and adaptive immune branches, and this short communication range is important to limit their effects to the appropriate cells. Although cytokines play an essential role in the host’s response to infection, they can also have deleterious effects. Thus, there is a fine balance between the positive and negative effects of cytokines on the host that is dictated by the duration, amount, and location of their expression [36].

RNA-seq has the major advantages of a higher dynamic range, precise quantification of transcripts, and comprehensive coverage of all expressed sequences in a given tissue [37]. Mastitis is regulated both by protein-coding genes and non-coding RNA. Fang et al. provided new insights into the genetic basis of mastitis and milk production in dairy cattle using a combination of GWAS and RNA-seq [38]. With advances in the development of multi-omics technology, the use of network analysis can reveal the potential central genes and genetic mechanisms regulating mastitis [39]. The udder health of dairy cattle can be monitored using blood and milk samples, as well as samples of udder tissue [40]. In this study, the expression profiles of mRNAs and non-coding RNAs were successfully constructed using peripheral blood samples of Xinjiang brown cattle, providing information on potential molecular markers and references for future studies on mastitis and mastitis resistance. Then, the PI3K-Akt pathway, which showed significant enrichment, is a classical signal transduction pathway involved in pro-survival and anti-apoptosis functions and plays an important role in a variety of biological processes, including cell proliferation and the invasion and metastasis of various cancers [41]. The focal adhesion pathway is associated both with structural and signal transduction functions and has been linked with bacterial recognition and subsequent signaling functions [42]. In interactions between the ECM and cell surface receptors, the ECM components provide not only tissue support but also participate in and contribute to many biological reactions [43]. The ECM also contains various secreted proteins, including cytokines, chemokines, and growth factors, which are potentially involved in immune cell regulation. Hence, these pathways and DEGs are likely to play important roles in the occurrence and development of mastitis.

It is well documented that ncRNAs play important roles in various physiological processes, including chromatin modification, transcriptional activation, and interference with transcription and translation [44]. The provision of miRNA-binding sites on lncRNAs allows the binding of miRNAs to lncRNAs, thus influencing the function of the latter. Meanwhile, according to the ceRNA hypothesis, miRNA can induce lncRNA degradation by combining with lncRNAs via RISC [45,46]. The concept of ceRNA has been widely applied in studies of the genetic mechanisms underlying key traits in cattle and sheep. For instance, Li et al. investigated the expression of lncRNAs in skeletal muscle tissues and constructed co-expression ceRNA networks in cattle and buffalo. The results showed that several lncRNAs (MSTRG.448330.7, MSTRG.300300.4, and MSTRG.203788.46) may contain potential binding sites for miR-1/206 and miR-133a [47]. Huang et al. established a ceRNA network and reported that some differentially expressed non-coding RNAs might be involved in the regulation of muscle traits. Liang et al. [48] identified 216 ceRNAs, including 26 lncRNAs, 27 miRNAs, and 64 mRNAs, in human mesenchymal stem cell (MSC) adipose differentiation at 0, 3, and 6 d. The mRNAs in the ceRNA network were found to be mainly associated with glucose homeostasis and the response to insulin stimulation, as well as many lipid metabolism-related pathways, such as Ras, hypoxia-inducible factor-1 (HIF-1), PI3K-Akt, and insulin signaling. Chen et al. constructed a ceRNA network using RNA-seq data and annotated potential candidate miRNAs and circRNAs involved in udder development in sheep, providing further insights into the influence of miRNAs and circRNAs on udder development in this species [49]. These studies not only identified many key molecular markers but also further demonstrated the integration of data from multiple sources for the construction of a global gene expression network, which could help us to understand the mechanisms underlying the formation of important traits [50]. Here, the differentially expressed ceRNA interaction networks of the control and clinical mastitis groups were successfully constructed using sequencing data. The key nodal mRNAs were found to be mainly enriched in pathways such as PI3K-Akt, cholinergic synapses, cell adhesion molecules, ion binding, cytokine receptor activity, and peptide receptor activity; these are consistent with the findings of previous studies of lncRNAs, circRNAs, and mRNAs. Moreover, the scope of candidate markers was further narrowed down, allowing efficient focus on key molecules. Notably, the lncRNA-miRNA-mRNA ternary co-expression network of *RHO*, *RCVRN*, *CSF1R*, *CAV3*, and *GATA4* genes is likely to be involved in the regulation of mastitis in Xinjiang brown cattle.

## 5. Conclusions

In conclusion, we compared the expressional features of mRNAs, lncRNAs, and circRNAs in the blood of healthy Xinjiang brown cattle and Xinjiang brown cattle with mastitis. In our results, genes such as TCONS_00211035, bta-miR-2415-3p, and CSF1R may play important roles in bovine mastitis. The result of circRNA/miRNA/mRNA and lncRNA/miRNA/mRNA interactions provides an important foundation for understanding the genetic process of bovine mastitis. In this study, a reference for research on mastitis resistance in Xinjiang brown cattle is provided.

## Data Availability

The raw data supporting the conclusions of this article will be made available by the authors on request.

## Supplementary Materials

The following supporting information can be downloaded at: https://www.mdpi.com/article/10.3390/genes15040465/s1, Table S1: Information on the RT-qPCR primer; Table S2: Total RNA concentration and quality data; Table S3: Quality statistics of sequencing data; Table S4: Sample mapping situation summary.
